# A new inactive conformation of SARS-CoV-2 main protease

**DOI:** 10.1107/S2059798322000948

**Published:** 2022-02-21

**Authors:** Emanuele Fornasier, Maria Ludovica Macchia, Gabriele Giachin, Alice Sosic, Matteo Pavan, Mattia Sturlese, Cristiano Salata, Stefano Moro, Barbara Gatto, Massimo Bellanda, Roberto Battistutta

**Affiliations:** a Department of Chemical Sciences, University of Padua, Via F. Marzolo 1, 35131 Padova, Italy; b Department of Pharmaceutical and Pharmacological Sciences, University of Padua, Via F. Marzolo 5, 35131 Padova, Italy; c Molecular Modeling Section, Department of Pharmaceutical and Pharmacological Sciences, University of Padua, Via F. Marzolo 5, 35131 Padova, Italy; d Department of Molecular Medicine, University of Padua, Via Gabelli 63, 35121 Padova, Italy; e Institute of Biomolecular Chemistry of CNR, Padua Unit, Via F. Marzolo 1, 35131 Padova, Italy

**Keywords:** SARS-CoV-2, main protease, COVID-19, M^pro^, crystal structure, inactive conformation

## Abstract

The SARS-CoV-2 main protease (M^pro^) has a pivotal role in mediating viral genome replication and transcription of the coronavirus, making it a promising target for drugs against the COVID-19 pandemic. Here, a crystal structure is presented in which M^pro^ adopts an inactive state that has never been observed before, called new-inactive. It is shown that the oxyanion loop, which is involved in substrate recognition and enzymatic activity, adopts a new catalytically incompetent conformation and that many of the key interactions of the active conformation of the enzyme around the active site are lost. Solvation/desolvation energetic contributions play an important role in the transition from the inactive to the active state, with Phe140 moving from an exposed to a buried environment and Asn142 moving from a buried environment to an exposed environment. In new-inactive M^pro^ a new cavity is present near the S2′ subsite, and the N-terminal and C-terminal tails, as well as the dimeric interface, are perturbed, with partial destabilization of the dimeric assembly. This novel conformation is relevant both for comprehension of the mechanism of action of M^pro^ within the catalytic cycle and for the successful structure-based drug design of antiviral drugs.

## Abstract 2

A new inactive conformation of SARS-CoV-2 main protease that is relevant to comprehension of the catalytic cycle and for structure-based drug-design approaches is reported.

## 1. Introduction

To face the global COVID-19 pandemic, besides prevention via the use of vaccines, it is also essential to develop targeted therapeutic options for patients infected by the SARS-CoV-2 betacoronavirus. In general, one of the most promising classes of antiviral drug candidates are protease inhibitors, small molecules that are able to inhibit enzymes involved in virus replication within the cell. Very low sequence identity with human proteases and distinct cleavage-site specificities suggest that viral enzymes can be inhibited with very low associated toxic effects (‘off-target’ effects), if any. Indeed, protease inhibitors have already been efficient in the treatment of viral pathogens such as hepatitis C virus (Pol & Corouge, 2014 ▸) and human immunodeficiency virus (HIV; Skwarecki *et al.*, 2021 ▸). In coronaviruses, the main protease, M^pro^, is a cysteine peptidase that is essential for the replication cycle of positive-sense, single-stranded RNA coronaviruses (Xia & Kang, 2011 ▸), including SARS-CoV-2. It is also known as 3C-like protease or 3CL^pro^ from the similarity of its active site and its substrate specificity to those of the picornavirus 3C protease (Anand *et al.*, 2002 ▸). M^pro^ is involved in the proteolytic processing of the two overlapping polyproteins pp1a and pp1ab, with the formation of individual mature nonstructural proteins (Snijder *et al.*, 2016 ▸), and as such it is a validated antiviral drug target (Dai *et al.*, 2020 ▸; Günther *et al.*, 2021 ▸; Ullrich & Nitsche, 2020 ▸). Currently, there are at least two SARS-CoV-2 M^pro^ inhibitors in phase I clinical trials as candidates with potent antiviral activity: the orally administered PF-07321332 (Pavan *et al.*, 2021 ▸) and the intravenously administered PF-00835231 (Ahmad *et al.*, 2021 ▸).

SARS-CoV-2 M^pro^ (nsp5), a 306-amino-acid poly­peptide of molecular weight 33.8 kDa (Wu *et al.*, 2020 ▸), shares 96% sequence identity and a very similar 3D structure with SARS-CoV M^pro^ [0.53 Å r.m.s.d. between PDB entries 6y2e (Zhang *et al.*, 2020 ▸) and 2bx4 (Tan *et al.*, 2005 ▸)]. Very similar 3D structures have also been found for other coronaviral M^pro^s such as those from Porcine transmissible gastroenteritis virus (TGEV), which was the first structure of a coronaviral M^pro^ (Anand *et al.*, 2002 ▸), Human coronavirus (HCoV) strain 229E (Anand *et al.*, 2003 ▸), Infectious bronchitis virus (IBV; Xue *et al.*, 2008 ▸) and MERS-CoV (Ho *et al.*, 2015 ▸). This structural similarity, which is particularly relevant around the active site, leads to the possibility of the development of pan-coronaviral drugs.

**Figure 1:**
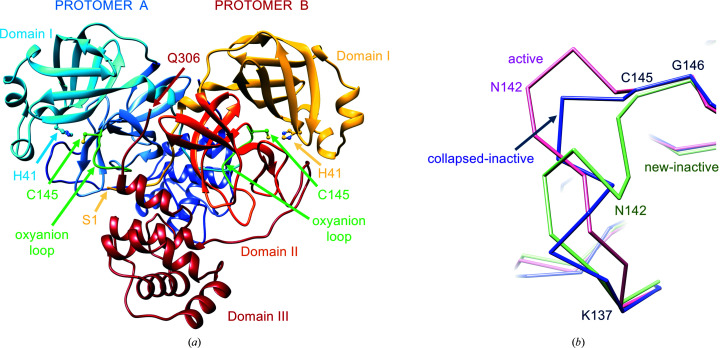
SARS-CoV-2 M^pro^ architecture, free form (PDB entry 6y2e). (*a*) Dimeric assembly of the protease with the main structural features discussed in the text highlighted. Protomer *A* is in blue-based colors and protomer *B* is in yellow/red-based colors. The two oxyanion loops and the two catalytic cysteines 145 are shown in green. (*b*) Comparison between different oxyanion-loop conformations of M^pro^: active in SARS-CoV-2 M^pro^ (PDB entry 6y2e) in pink, collapsed-inactive in SARS-CoV M^pro^ (PDB entry 1uj1 chain *B*) in magenta and new-inactive in SARS-CoV-2 M^pro^ (this work) in green.

M^pro^ exists in an equilibrium between a monomer and a homodimer (with the two protomers roughly perpendicularly oriented; Fig. 1 ▸ *a*), with an apparent *K*_d_ of between 0.8 and 14 µ*M* for the SARS-CoV enzyme, depending on the experimental conditions (Chen *et al.*, 2006 ▸). For SARS-CoV-2 M^pro^, the *K*_d_ has been estimated to be 2.5 µ*M* by analytical ultracentrifugation (Zhang *et al.*, 2020 ▸) and 0.14 µ*M* by native mass spectrometry (El-Baba *et al.*, 2020 ▸). Unlike 3C protease, only the SARS-CoV M^pro^ dimer shows enzymatic activity (Anand *et al.*, 2002 ▸) and the correct shape of the substrate-binding site, particularly of the S1 subsite; the correct conformation for productive catalytic events is linked to the dimerization process. It has been proposed that the dimerization process has a direct regulatory role of the activity of M^pro^ during the coronaviral replication process (Hsu *et al.*, 2005 ▸; Li *et al.*, 2016 ▸). Given the high structural similarity, particularly at the dimeric interface, it was reasoned that dimerization of the enzyme is also necessary for the catalytic activity of SARS-CoV-2 M^pro^ (Zhang *et al.*, 2020 ▸),

Each M^pro^ protomer is composed of three structural domains (Fig. 1 ▸ *a*; Anand *et al.*, 2002 ▸). The chymotrypsin-like and 3C protease-like β-barrel domains I (residues 1–99) and II (residues 100–182) directly control the catalytic event. The substrate-binding site is between these two domains and comprises several subsites for substrate binding (from S1 to S6 and from S1′ to S3′), corresponding to the P1–P6 and P1′–P3′ amino-acid positions of the substrates (according to the convention P6–P5–P4–P3–P2–P1↓P1′–P2′–P3′, where ↓ indicates the hydrolyzed peptide bond; Anand *et al.*, 2003 ▸). Enzymatic proteolysis by SARS-CoV-2 M^pro^ at the 11 cleavage sites on the viral polyprotein occurs on the C-terminal side of a conserved glutamine in position P1, with the most common consensus sequence being Leu-Gln↓(Ser/Ala), indicating that specificity is determined mostly by the P2, P1 and P1′ positions (Ullrich & Nitsche, 2020 ▸). Glutamine in position P1 is fully conserved not only for SARS-CoV-2 but also in substrates of SARS-CoV and MERS-CoV. Prime recognition sites at the C-terminus of P1′ are not conserved. M^pro^ subsites S4, S2, S1 and S1′ have been identified as the most relevant subsites for substrate binding, with regions in the S5, S4 and S2 sites showing considerable conformational flexibility upon binding different chemical groups (Kneller, Galanie *et al.*, 2020 ▸). The chymotrypsin-like fold, including domains I and II, is connected by a 16-residue flexible loop to the extra α-helical domain III (residues 198–306; Fig. 1 ▸ *a*). Domain III is absent in other RNA virus 3C-like proteases and plays a key role in enzyme dimerization and activity regulation of M^pro^ (Anand *et al.*, 2002 ▸; Shi & Song, 2006 ▸).

At variance with the classical catalytic triad of chymotrypsin-like proteases, coronaviral M^pro^ has a catalytic dyad, consisting of His41 and Cys145 in SARS-CoV-2 (Fig. 1 ▸ *a*); a conserved water molecule occupies a position analogous to that of the side chain of the third member of the catalytic triad (for instance, aspartate in chymotrypsin and asparagine in papain) and forms hydrogen bonds to the side chains of His41, His164 and Asp187. It has been proposed that this conserved water is involved in the catalytic event (Anand *et al.*, 2002 ▸).

A key role in the proper function of the enzyme is also played by the N-finger (residues 1–7) as the N-terminal tail of one protomer interacts and stabilizes the binding site (S1 subsite) of the other protomer (Verschueren *et al.*, 2008 ▸). Indeed, deletion of the N-finger hampers dimerization in solution and abolishes the proteolytic activity. Both the N-finger and the C-terminus are results of the autoproteolytic processing of M^pro^. Accordingly, in the mature dimeric enzyme both termini of one protomer face the active site of the other.

The important conserved residues Phe140, Leu141, Asn142 and Ser144 (SARS-CoV-2 numbering) are part of a structural element that is essential for a productive catalytic event, the so-called oxyanion loop comprising residues 138–145, which globally lines the binding site for glutamine P1. The central role of the oxyanion loop in the catalytic reaction mechanism of serine proteases and cysteine proteases has been extensively characterized (Frey & Hegeman, 2007 ▸). The correct positioning of the oxyanion hole, which is part of the oxy­anion loop (formed by the backbone of Gly143, Ser144 and Cys145 in SARS-CoV-2 M^pro^), is essential for stabilization of the transient tetrahedral acyl (oxyanion) transition state via the hydrogen-bond donor properties of the amides (Anand *et al.*, 2002 ▸; Lee *et al.*, 2020 ▸; Verschueren *et al.*, 2008 ▸). In the known crystal structures of SARS-CoV and SARS-CoV-2 M^pro^, the oxyanion loop adopts essentially the same ‘active’ conformation; here, we take PDB entry 6y2e as a reference for this conformation (Douangamath *et al.*, 2020 ▸; Jin, Du *et al.*, 2020 ▸; Jin, Zhao *et al.*, 2020 ▸; Zhang *et al.*, 2020 ▸). A specific conformation is defined to be active when the amino acids known to participate in the chemical reaction catalyzed by the enzyme are properly positioned and oriented for the reaction to proceed. We also term this conformation catalytically competent.

Variations from the active conformation of the oxyanion loop are found in a few forms of the enzyme, which were consequently considered to be inactive or catalytically incompetent, as in protomer *B* of SARS-CoV M^pro^ (PDB entries 1uj1 and 1uk2; Yang *et al.*, 2003 ▸), in the monomeric R298A mutant of SARS-CoV M^pro^ (PDB entry 2qcy; Shi *et al.*, 2008 ▸) and in the C172A mutant of 3C^pro^ from the picornavirus hepatitis A virus (Allaire *et al.*, 1994 ▸), as well as in IBV 3CL^pro^ (PDB entries 2q6f and 2q6d; Xue *et al.*, 2008 ▸). In the inactive monomeric R298A mutant (PDB entry 2qcy), the region of the oxyanion loop, Ser139-Phe140-Leu141, is converted into a short 3_10_-helix. In PDB entry 1uj1 (SARS-CoV M^pro^ crystallized at pH 6) the oxyanion loop of one of the two protomers exists in a ‘collapsed’ conformation (similar to that found in PDB entry 2qcy), which is considered to be catalytically incompetent, in which the hydrogen bond between Glu166 and His172 that is important for activity is broken (Yang *et al.*, 2003 ▸). In the following, we will refer to these two inactive conformations with similar oxyanion-loop conformations as collapsed-inactive (Fig. 1 ▸ *b*).

In the vast majority of SARS-CoV and SARS-CoV-2 M^pro^ crystal structures, the dimer is crystallographic (Jaskolski *et al.*, 2021 ▸); that is, there is only one molecule in the asymmetric unit and therefore the two protomers are perfectly identical. In the very few inactive structures, apart from the artificially induced monomeric forms, the dimer is formed by two different molecules present in the asymmetric unit, one of which is in the inactive state and the other of which is in the active state. Based on molecular-dynamics simulations coupled to activity data in solution, it was suggested that only one protomer at a time is active in the dimer (Chen *et al.*, 2006 ▸).

Here, we describe a new inactive structure (called new-inactive) of the main protease of SARS-CoV-2 that is clearly distinct from both the active and the known collapsed-inactive structures, with an oxyanion-loop conformation that is very different from those previously described (Fig. 1 ▸ *b*). In Section 4, we argue that this conformation has an important functional role as part of the catalytic cycle of coronaviral M^pro^.

## 2. Materials and methods

### 2.1. Recombinant protein production and purification

The plasmid PGEX-6p-1 encoding SARS-CoV-2 M^pro^ (Zhang *et al.*, 2020 ▸) was a generous gift from Professor Rolf Hilgenfeld, University of Lübeck, Lübeck, Germany. Recombinant protein production and purification were adapted from Zhang *et al.* (2020 ▸) (where the structure of M^pro^ in the active form was presented; PDB entry 6y2e). The expression plasmid was transformed into *Escherichia coli* strain BL21 (DE3) and then precultured in YT medium at 37°C (100 µg ml^−1^ ampicillin) overnight. The preculture was used to inoculate fresh YT medium supplemented with antibiotic and the cells were grown at 37°C to an OD_600_ of 0.6–0.8 before induction with 0.5 m*M* isopropyl β-d-1-thiogalactopyranoside (IPTG). After 5 h at 37°C, the cells were harvested by centrifugation (5000*g*, 4°C, 15 min) and frozen. The pellets were resuspended in buffer *A* (20 m*M* Tris, 150 m*M* NaCl pH 7.8) supplemented with lysozyme, DNase I and PMSF for lysis. The lysate was clarified by centrifugation at 12 000*g* at 4°C for 1 h and loaded onto a HisTrap HP column (GE Healthcare) equilibrated with 98% buffer *A*/2% buffer *B* (20 m*M* Tris, 150 m*M* NaCl, 500 m*M* imidazole pH 7.8). The column was washed with 95% buffer *A*/5% buffer *B*, and His-tagged M^pro^ was then eluted with a linear gradient of imidazole from 25 to 500 m*M*. Pooled fractions containing the target protein were subjected to buffer exchange with buffer *A* using a HiPrep 26/10 desalting column (GE Healthcare). Next, PreScission protease was added to remove the C-terminal His tag (20 µg of PreScission protease per milligram of target protein) at 12°C overnight. The protein solution was loaded onto a HisTrap HP column connected to a GSTrap FF column (GE Healthcare) equilibrated in buffer *A* to remove the GST-tagged Pre­Scission protease, the His tag and the uncleaved protein. M^pro^ was finally purified using a Superdex 75 prep-grade 16/60 SEC column (GE Healthcare) equilibrated with buffer *C* (20 m*M* Tris, 150 m*M* NaCl, 1 m*M* EDTA, 1 m*M* DTT pH 7.8). Fractions containing the target protein with high purity were pooled, concentrated to 25 mg ml^−1^ and flash-frozen in liquid nitrogen for storage in small aliquots at −80°C.

### 2.2. Protein characterization and enzymatic kinetics

The correctness of the M^pro^ DNA sequence was verified by sequencing the expression plasmid. The molecular mass was determined as follows: recombinant SARS-CoV-2 M^pro^, diluted in 50% acetonitrile with 0.1% formic acid, was analyzed by direct infusion electrospray ionization (ESI) on a Xevo G2-XS QTOF mass spectrometer (Waters). The detected species displayed a mass of 33 796.64 Da, which very closely matches the value of 33 796.81 Da calculated from the theoretical full-length protein sequence (residues 1–306). A representative ESI-MS spectrum is shown in Supplementary Fig. S1. To characterize the enzymatic activity of our recombinant M^pro^, we adopted a FRET-based assay using the substrate 5-FAM-AVLQ↓SGFRK(DABCYL)K (Proteogenix). The assay was performed by mixing 0.05 µ*M* M^pro^ with various concentrations of substrate (1–128 µ*M*) in a buffer composed of 20 m*M* Tris, 100 m*M* NaCl, 1 m*M* EDTA, 1 m*M* DTT pH 7.3. Fluorescence intensity (excitation at 485 nm and emission at 535 nm) was monitored at 37°C with a VictorIII microplate reader (Perkin Elmer). A calibration curve was created by measuring multiple concentrations (from 0.001 to 5 µ*M*) of free fluorescein in a final volume of 100 µl reaction buffer. Initial velocities were determined from the linear section of the curve, and the corresponding relative fluorescence units per time unit (ΔRFU s^−1^) were converted to the amount of cleaved substrate per time unit (µ*M* s^−1^) by fitting to the calibration curve of free fluorescein. The catalytic efficiency *k*_cat_/*K*_m_ was 4819 ± 399 s^−1^ *M* ^−1^, which is in line with literature data (Ma *et al.*, 2020 ▸; Zhang *et al.*, 2020 ▸).

### 2.3. Crystallization and data collection

A frozen aliquot of M^pro^ was thawed in ice, diluted in a 1:2 ratio with buffer *C* (20 m*M* Tris, 150 m*M* NaCl, 1 m*M* EDTA, 1 m*M* DTT pH 7.8) to a final concentration of 12.5 mg ml^−1^ and clarified by centrifugation at 16 000*g*. The inhibitors masitinib, manidipine, bedaquiline and boceprevir were dissolved in 100% DMSO to a concentration of 100 m*M*. The protein was crystallized both in the free form and in the presence of inhibitors by co-crystallization. In all cases, final crystal growth was obtained by microseeding starting from small crystals of the free enzyme. The protein in the free form was crystallized using the sitting-drop vapor-diffusion method at 18°C, mixing 1.0 µl M^pro^ solution with 1.0 µl precipitant solution [0.1 *M* MMT (dl-malic acid, MES and Tris base in a 1:2:2 molar ratio) pH 7.0, 25% PEG 1500] and 0.2 µl seed stock (diluted 1:500, 1:1000 or 1:2000 with precipitant solution) and equilibrating against a 300 µl reservoir of precipitant solution. Crystals appeared overnight and grew for 48 h after the crystallization drops had been prepared. In the case of co-crystallization, M^pro^ was incubated for 16 h at 8°C with a 13-fold molar excess of inhibitor (final DMSO concentration 5%). After incubation with masitinib, manidipine or bedaquiline, a white precipitate appeared and the solutions were clarified by centrifugation at 16 000*g*; as the protein concentration was essentially unchanged after centrifugation, we concluded that the precipitate is composed of the inhibitors, which are poorly soluble in water. The fact that the protein was later crystallized under the same conditions as described for the free form further confirmed that its concentration was not altered by the centrifugation process. For data collections, crystals were fished from the drops, cryoprotected by a quick dip into 30% PEG 400 (with 5 m*M* inhibitor in the case of co-crystals) and flash-cooled in liquid nitrogen. The crystals were monoclinic (space group *C*2), isomorphous to the crystals of the free enzyme (PDB entry 6y2e), with one monomer in the asymmetric unit; the dimer is formed by the crystallographic twofold axis.

### 2.4. Structure determination, refinement and analysis

Data were collected on beamlines ID23-2 and ID23-1 at the ESRF. Diffraction data integration and scaling were performed with *XDS* (Kabsch, 2010 ▸) and data reduction and analysis were performed with *AIMLESS* (Evans & Murshudov, 2013 ▸). Initially, structures were solved by molecular replacement (MR) with *Phaser* (McCoy *et al.*, 2007 ▸) in *Phenix* (Liebschner *et al.*, 2019 ▸) using PDB entries 6y2e and 5rel (M^pro^ in complex with PCM-0102340; Douangamath *et al.*, 2020 ▸) as search models. To limit MR model bias in critical zones (namely residues 139–144, 1–3 and the side chain of His163) we then performed new MR runs using PDB entry 6y2e without residues 139–144 and 1–3, and with an alanine instead of a histidine at position 163, as the search model. Only for co-crystallization experiments with boceprevir was electron density for the ligand clearly visible from the beginning of the refinement (Supplementary Figs. S2 and S3), and the three final structures, modeled from residues 1 to 306 (compared with the ‘new’ structure modeled to residue 301), are virtually identical to those deposited in the PDB (Fu *et al.*, 2020 ▸). In all of the other cases, no electron density indicating the presence of the inhibitors masitinib, manidipine or bedaquiline in the active site (or elsewhere) was detectable. For four structures, it was possible to efficiently model residues 139–144, 1–3 and the side chain of His163 in ‘new’ conformations. The final structures were obtained by alternating cycles of manual refinement with *Coot* (Emsley *et al.*, 2010 ▸) and automatic refinement with *phenix.refine* (Afonine *et al.*, 2012 ▸). At the end, the model was submitted to *phenix.ensemble_refinement* (Burnley *et al.*, 2012 ▸) with default parameters. Data-collection and refinement statistics for the structure obtained by a co-crystallization experiment with masitinib (which was not visible in the final electron density) are reported in Table 1 ▸. Secondary-structure analysis was performed with *DSSP* (Kabsch & Sander, 1983 ▸; Touw *et al.*, 2015 ▸). Local energetic frustration analysis was performed with the *Frustratometer* server (http://frustratometer.qb.fcen.uba.ar; Parra *et al.*, 2016 ▸). Interface analysis was performed using *PISA* (Krissinel & Henrick, 2007 ▸).

### 2.5. Molecular modeling

The majority of the computational work was performed on a Linux desktop workstation (Intel Xeon CPU E5-1620 3.60 GHz) running Ubuntu 16.04 LTS. Molecular-dynamics trajectories were collected on a heterogeneous Nvidia GPU cluster composed of 20 GPUs with models spanning from GTX1080 to RTX2080Ti. For structure preparation, coordinates of the active conformation of SARS-CoV-2 M^pro^ were retrieved from the Protein Data Bank (PDB entry 6y2e). Coordinates for both the active and the new-inactive conformation were processed with the aid of the *Molecular Operating Environment* (*MOE*) 2019.01 (Chemical Computing Group) structure-preparation tool. Initially, the functional unit of the protease (the dimeric form) was restored by applying a symmetric crystallographic transformation to each asymmetric unit. Residues with alternate conformations were assigned to the highest occupancy alternative. Moreover, missing residues that are present in the primary sequence were added using the *MOE* Loop Modeler tool. The *MOE* Proton­ate3D tool was used to assign the most probable protonation state to each residue (pH 7.4, *T* = 310 K, i.f. = 0.154). Partial charges were then assigned using the AMBER10 force field and H atoms were energy-minimized until the gradient was below 0.1 kcal mol^−1^ Å^−2^. Finally, ions and all co-crystallized molecules except for water were removed before saving the structures. The system setup for the MD simulations was carried out using the *antechamber*, *parmchk* and *tleap* software implemented in the *AmberTools*14 suite (Case *et al.*, 2005 ▸). AMBER ff14SB (Maier *et al.*, 2015 ▸) was adopted for system parametrization and attribution of partial charges. Protein structures were explicitly solvated in a rectangular prismatic TIP3P (Jorgensen *et al.*, 1983 ▸) periodic water box with borders placed at a distance of 15 Å from any protein atom. Na^+^ and Cl^−^ ions were added to neutralize the system until a salt concentration of 0.154 *M* was reached. MD simulations were then performed using *ACEMD*3 (Harvey *et al.*, 2009 ▸), which is based upon an OpenMM 7.4.2 engine (Eastman *et al.*, 2017 ▸). Initially, 1000 steps of energy minimization were executed using the conjugate-gradient algorithm. A two-step equilibration procedure was then carried out: the first step consisted of a 1 ns canonical ensemble (NVT) simulation with 5 kcal mol^−1^ Å^−2^ harmonic positional constraints applied to each protein atom, while the second step consisted of a 1 ns isothermal–isobaric (NPT) simulation with 5 kcal mol^−1^ Å^−2^ harmonic positional constraints applied only to protein C^α^ atoms. The production phase consisted of three independent MD replicas for each protein conformation. Each simulation had a duration of 1 µs and was performed using the NVT ensemble at a constant temperature of 310 K with a timestep of 2 fs. For both the equilibration and the production stage, the temperature was maintained constant using a Langevin thermostat. During the second step of the equilibration stage, the pressure was maintained at a fixed value of 1 atm with a Monte Carlo barostat. MD trajectories were aligned using protein C^α^ atoms from the first trajectory frame as a reference, wrapped into an image of the system under periodic boundary conditions (PBC), and subsequently saved using a 200 ps interval between each frame and removing any ions and water molecules using *Visual Molecular Dynamics* 1.9.2 (*VMD*; Humphrey *et al.*, 1996 ▸). The protein radius of gyration (*R*_g_), the root-mean-square deviation (r.m.s.d.) and the root-mean-square fluctuation (r.m.s.f.) of atomic positions along the trajectory were calculated for protein C^α^ atoms exploiting the MDAnalysis (Gowers *et al.*, 2016 ▸; Michaud-Agrawal *et al.*, 2011 ▸) Python module. Secondary-structure analysis was carried out with the *STRIDE* package (Frishman & Argos, 1995 ▸) as implemented in *VMD* 1.9.2. The collected data were then plotted using the Matplotlib Python library (Hunter, 2007 ▸).

Furthermore, two classic MD simulations were performed on the complexes obtained by superposing the coordinates of peptide ligands from PDB entries 2q6g and 7khp on the new-inactive conformation of SARS-CoV-2 M^Pro^ using *MOE* 2019.01. For each peptide–ligand complex, a two-stage equilibration protocol followed by a single productive simulation was carried out. The first equilibration step consisted of a 0.1 ns canonical ensemble (NVT) simulation with 5 kcal mol^−1^ Å^−2^ harmonic positional constraints applied to each protein atom, while the second equilibration step consisted of a 0.5 ns isothermal–isobaric (NPT) simulation with 5 kcal mol^−1^ Å^−2^ harmonic positional constraints applied only to protein C^α^ atoms. For both equilibration simulations, the temperature was maintained constant (*T* = 310 K) using a Langevin thermostat, while during the second equilibration stage the pressure was kept at a constant value of 1 atm using a Monte Carlo barostat. The productive simulation was carried out for 10 ns in the NVT ensemble (*T* = 310 K).

## 3. Results

### 3.1. Identification of a new-inactive conformation of M^pro^

In a campaign to obtain structural insights into SARS-CoV-2 M^pro^, we analyzed 27 different data sets to determine crystal structures of M^pro^ in complex with different inhibitors, among which were masitinib, manidipine and bedaquiline (Ghahremanpour *et al.*, 2020 ▸). As ‘positive’ controls (*i.e.* structures that were already known), we considered ligand-free M^pro^ and M^pro^ in complex with the known α-ketoamide covalent reversible inhibitor boceprevir, an approved HCV drug that is also able to bind to SARS-CoV-2 M^pro^ (Fu *et al.*, 2020 ▸). M^pro^ samples were produced and crystallized in parallel, with very similar experimental procedures, analogous to those of the active enzyme (PDB entry 6y2e; Zhang *et al.*, 2020 ▸; see Section 2). Almost all tested crystals were monoclinic (space group *C*2, with unit-cell parameters *a* ≃ 113.1, *b* ≃ 54.7, *c* ≃ 44.8 Å, α = 90.0, β ≃ 101.3, γ = 90.0°), isomorphous to the crystals of the free active enzyme (PDB entry 6y2e; Zhang *et al.*, 2020 ▸) and to most of the deposited M^pro^ structures, signifying the same crystal contacts. After successful molecular replacement and a first round of refinement, in most cases (including the complex with boceprevir) electron density was clearly visible for the entire sequence, indicating a protein matrix with a very similar structure to the search models (PDB entries 6y2e and 5rel; Douangamath *et al.*, 2020 ▸). However, there were a significant number of cases, around ten, in which the electron density was of much lower quality or was even absent in particular portions of the protein, namely residues 139–144 of the oxyanion loop, residues 1–3 of the N-finger and the side chain of His163 in the S1 specificity subsite, all of which are residues that are part of the active site. To cope with the known molecular-replacement bias problem and to correctly rebuild the ambiguous parts, we performed new MR runs using PDB entry 6y2e deprived of residues 139–144 and 1–3, and with an alanine instead of a histidine at position 163 (to remove the His side chain), as a search model. This allowed us to confirm perturbations in the conformation of the selected areas for ten structures, while clear electron density was visible for the remaining cases with the oxyanion loop unambiguously in the active conformation (Supplementary Figs. S2 and S3). In some cases, the electron density was so poor that the tracing of the chain was very problematic, and it was not possible to reliably rebuild the mobile zones entirely (Supplementary Fig. S2*b* ). For four structures, it was possible to efficiently model residues 139–144, residues 1–3 and the side chain of His163 in ‘new’ conformations (‘new’ because there are no equivalents in M^pro^ structures deposited in the PDB) that differ from the active conformations and also from the collapsed-inactive conformations, including PDB entry 2qcy, where the oxyanion loop adopts a 3_10_-helix conformation (Supplementary Fig. S2*c* ). In this regard, comprehensive analyses of the available SARS-CoV and SARS-CoV-2 M^pro^ crystal structures have recently appeared in the literature (Behnam, 2021 ▸; Brzezinski *et al.*, 2021 ▸; Jaskolski *et al.*, 2021 ▸; Wlodawer *et al.*, 2020 ▸). In no case was a conformation analogous to that presented here described, confirming our assessment of a new-inactive state. The most relevant structures discussed here are reported in Supplementary Table S1.

In summary, we found three different conformational states for the oxyanion loop: active (Supplementary Fig. S2*a* ), flexible (*i.e.* with poor electron density; Supplementary Fig. S2*b* ) and, strikingly, a new-inactive state (Supplementary Fig. S2*c* ). A comparison of the known active and collapsed-inactive conformations with the new-inactive conformation presented here is shown in Fig. 1 ▸(*b*).

The new-inactive structures were derived solely from crystals obtained using M^pro^ pre-incubated with the inhibitors masitinib, manidipine or bedaquiline, but in no case was electron density indicating the presence of the inhibitors detected. This is explainable by the medium/high IC_50_ (in the range 2.5–19 µ*M*; Drayman *et al.*, 2021 ▸; Ghahremanpour *et al.*, 2020 ▸) and the very low aqueous solubility of the molecules (when inhibitors in 100% DMSO were added to the protein solution, visible white precipitates appeared). It is tempting to speculate that the presence of these inhibitors in solution plays a role in favoring the selection of the new-inactive conformation by the crystallization process. Some structures of crystals from co-crystallization experiments with masitinib or manidipine, again without any evidence for the presence of the ligand in the binding site, show the oxyanion active conformation. This indicates that these molecules, although favoring the new state, are not strict determinants for its formation. In the free form of the enzyme (from crystallization experiments with no ligands), we obtained structures with very clear electron density for the oxyanion loop, as shown in Supplementary Fig. S2(*a*), with low local *B* factors in the refined model, but also structures with a very ‘destabilized’, mobile oxyanion loop, as in Supplementary Fig. S2(*b*), with much higher *B* factors in the final model. This suggests that the high flexibility of the oxyanion loop is an intrinsic property of the free enzyme and is not artificially induced by the presence of ligands in the crystallization experiments.

**Figure 2:**
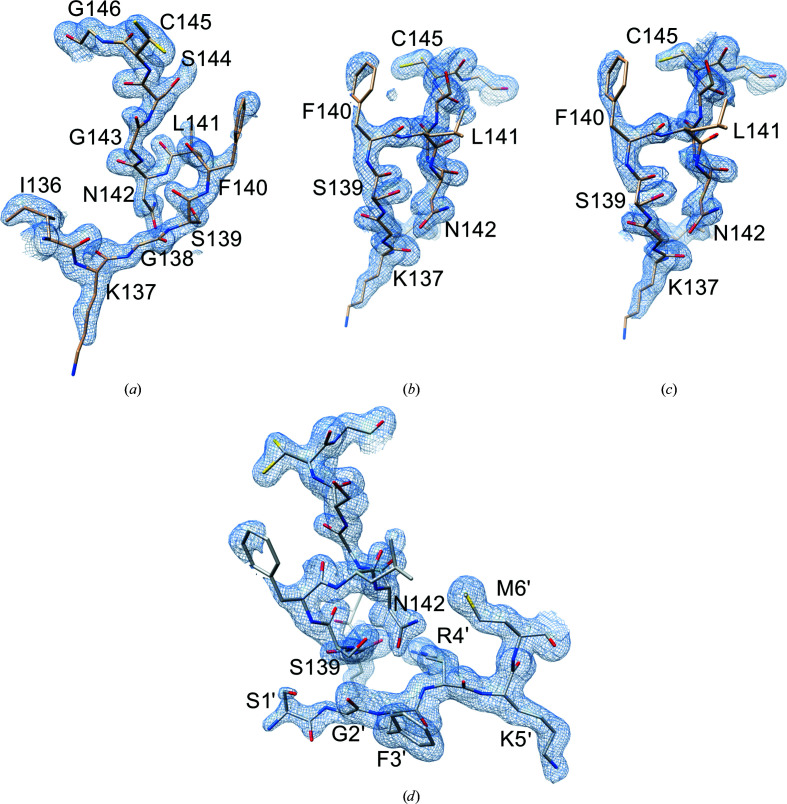
Final electron densities for the most relevant regions of new-inactive M^pro^. 2*F*_o_ − *F*_c_ maps contoured at the 1.0σ level are shown. (*a*) and (*b*) show two views of the final electron density for the oxyanion loop in the new conformation. Leu141 and the solvent-exposed Phe140 and Lys137 side chains have incomplete densities indicating various degrees of flexibility. (*c*) Simulated-annealing omit map (oxyanion-loop residues 138–146 were omitted) viewed as in (*b*). (*d*) Electron density in the inter-protomer (intra-dimer) interaction area between the oxyanion loop of one protomer and the N-finger of the other protomer (residues Ser1′–Met6′).

Here, we describe only one of the structures of M^pro^ determined in the new-inactive conformation, which was obtained by co-crystallization experiments with masitinib (no relevant differences exist among the four new-inactive M^pro^ structures). Data-collection and final model statistics are reported in Table 1 ▸; final electron densities for the most relevant regions discussed in the text are shown in Fig. 2 ▸. Unlike in other inactive structures of the enzyme, in which only one protomer adopts the inactive conformation, the dimeric arrangement of the new structure is due to a crystallographic symmetric axis, and the two subunits are therefore identical and both inactive.

### 3.2. The oxyanion loop adopts a novel inactive conformation

**Figure 3:**
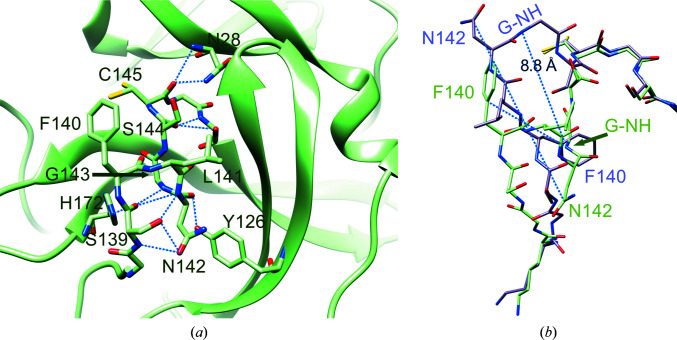
Details of the hydrogen-bond interactions in the oxyanion region of new-inactive M^pro^. (*a*) The new conformation of the oxyanion loop is stabilized by several backbone hydrogen bonds (blue dashed lines) as described in the main text. The side chain of catalytic Cys145 has a double conformation. (*b*) Comparison between the new-inactive (green) and active (light magenta; PDB entry 6y2e) oxyanion loops. There are large movements (blue dashed lines) of the side chains of Asn142 and Phe140. In the new-inactive conformation, Asn142 moves from an exposed position with an ASA of 153.74 Å^2^ to a buried position with an ASA of 49.00 Å^2^ and Phe140 moves from a buried position with an ASA of 14.79 Å^2^ to an exposed position with an ASA of 143.29 Å^2^. Gly143 NH (G-NH) of the oxyanion hole, which is involved in the stabilization of the tetrahedral intermediate, moves 8.8 Å away.

The most striking property of the new structure is the significantly different conformational state of the oxyanion loop (Figs. 1 ▸ and 3 ▸), which is essential for stabilization of the tetrahedral acyl (oxyanion) transition state during the catalytic cycle. The loop backbone is stabilized by many hydrogen bonds in the new state (Fig. 3 ▸ *a*). According to the *DSSP* standardized secondary-structure assignment (Kabsch & Sander, 1983 ▸; Touw *et al.*, 2015 ▸), in the new oxyanion loop there are two consecutive ‘3-turns’ (β-turns) with hydrogen bonds between Leu141 CO and Ser144 NH and between Ser144 CO and Ser147 NH. This region is further stabilized by a ‘4-turn’ (α-turn) with a hydrogen bond between Ser139 CO and Gly143 NH. *DSSP* does not recognize any 3_10_-helical segments in the oxyanion loop (as present in the inactive PDB entry 2qcy).

There are other hydrogen bonds involving the backbone that stiffen the oxyanion loop: between Cys145 CO and Asn28 NH, between His163 CO and Gly146 NH and between Ser147 CO and His163 NH (Fig. 3 ▸ *a*). As a result, the new conformation appears to be quite stable and rigid, as confirmed by the good quality of the local electron density (Fig. 2 ▸ and Supplementary Fig. S2*c* ).

To analyze the energetics of the local contacts, we performed an energetic frustration analysis (Parra *et al.*, 2016 ▸) on the active and new-inactive conformations. The concept of local frustration in protein structure refers to possible residual energetic conflicts in local interactions in folded proteins, using a ‘frustration index’ that measures how favorable a particular contact is relative to the set of all possible contacts in that location (Chen *et al.*, 2020 ▸). The ‘principle of minimal frustration’ assumes that proteins find their native state by minimizing the internal energetic conflicts within their polypeptide chain (Bryngelson & Wolynes, 1987 ▸). The degree of frustration is therefore dependent on the type of amino acids involved in the interaction. Local violations of this principle have been recognized to be important to exert the proper biological functions, specifically around the active sites of protein enzymes (Freiberger *et al.*, 2019 ▸). Analysis of the local configurational frustration of the most interesting contacts around the active site of active and new-inactive M^pro^ is shown in Supplementary Table S2. In both conformations, the catalytic Cys145 is a minimally frustrated ‘hub’ (here we call a position with ≥10 minimally frustrated interactions a minimally frustrated hub), with a small prevalence of interactions in the active conformation. On the other hand, the difference for Phe140 is striking: eight minimally frustrated interactions are present in active M^pro^ (where it is buried in a hydrophobic pocket) as opposed to no interactions in new-inactive M^pro^ (where it is solvent-exposed). Differences between the two structures are also evident for other amino acids of the oxyanion loop, namely Leu141, Gly143 and Ser144, indicating their diverse involvement in the local energetic contributions. The oxyanion loop of inactive M^pro^ has a larger number of minimally frustrated interactions with Cys117. This residue is a minimally frustrated hub in both conformations; however, given the higher number of minimally frustrated interactions in new-inactive M^pro^ (18 versus ten), Cys117 seems to play an important role in the stabilization of the new-inactive conformation. Internal to the oxyanion loop there is also a highly frustrated (unfavorable) interaction involving Leu141, with Ser139 in new-inactive M^pro^ and with Ser144 in active M^pro^. This suggests that Leu141 may be important in switching between the two conformations.

### 3.3. Many key interactions of the active enzyme are lost in new-inactive M^pro^

**Figure 4:**
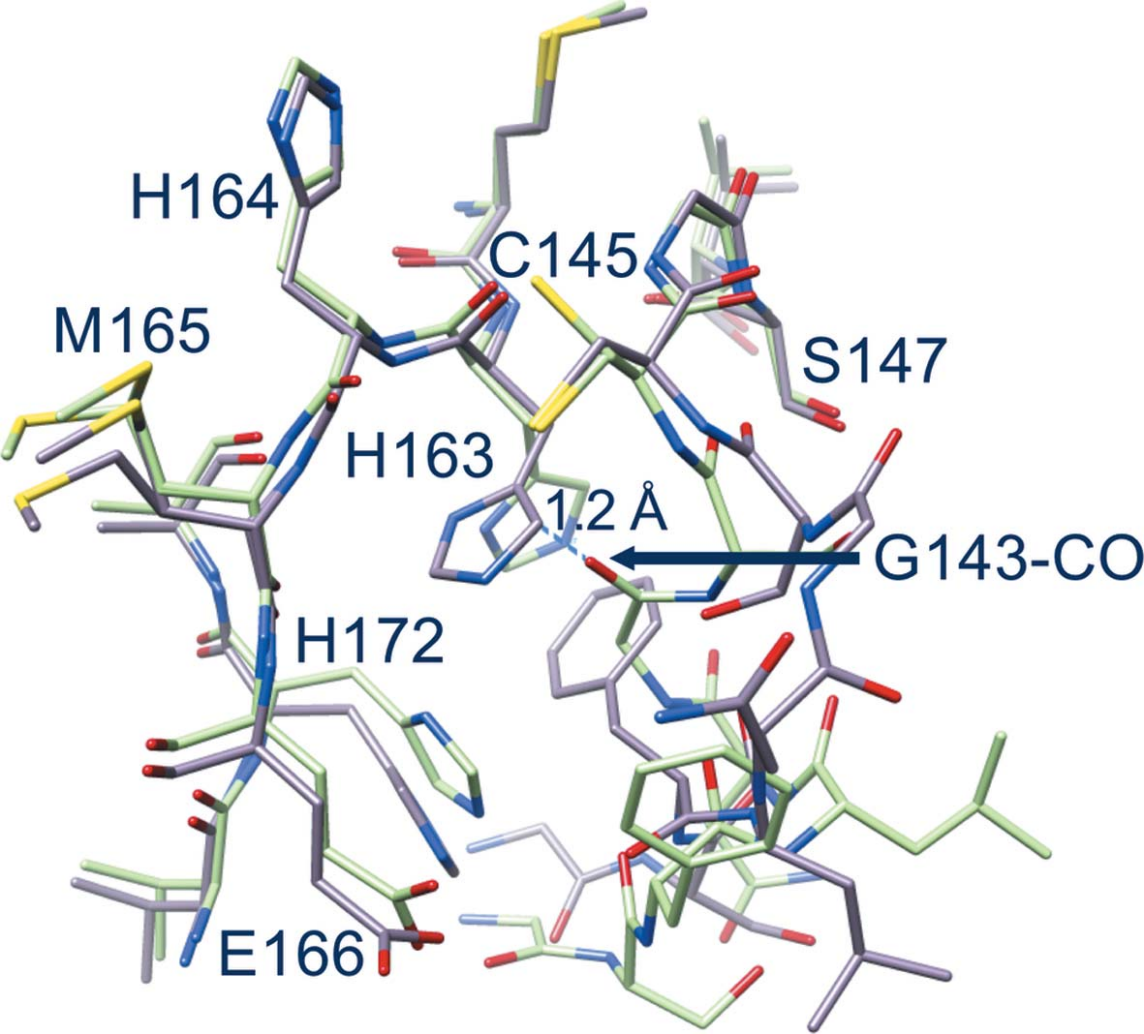
Comparison between new-inactive (green) and active (light magenta) M^pro^. In the new structure the side chain of His163 rotates away to avoid steric clashes with the oxyanion loop: in the active conformation (PDB entry 6y2e) the His163 side chain would be 1.2 Å from the new position of Gly143 CO. Note also the movement of His172.

The correct location of Phe140, Leu141, Asn142, Ser144, Tyr161, His163, Met165, Glu166 and His172 (as seen in the active PDB entry 6y2e, for instance) is an absolute requirement for the reaction catalyzed by M^pro^ to properly proceed, with special reference to stabilization of the tetrahedral acyl-intermediate (Anand *et al.*, 2002 ▸; Lee *et al.*, 2020 ▸; Verschueren *et al.*, 2008 ▸). Notably, all of these residues are conserved among known coronaviral M^pro^s, underlining their importance. In the new structure of M^pro^ most of these residues move away from the ‘active location’: Phe140, Leu141, Asn142 and Ser144 because of displacement of the oxyanion loop (Fig. 3 ▸ *b*) and His163 and His172 because of rotation of their side chains (Fig. 4 ▸).

Specifically, Asn142 C^α^ and the side chain of Phe140 are remarkably shifted from the active position by 9.8 and 7.5 Å, respectively (Fig. 3 ▸ *b*). Phe140, which is buried in a hydrophobic cleft in active M^pro^ with as accessible surface area (ASA) of 14.79 Å^2^), is now exposed to the solvent (ASA 143.29 Å^2^), while Asn142, which is exposed in active M^pro^ (ASA 153.74 Å^2^), is now buried (ASA 49.00 Å^2^). The side chain of Asn142 is locked in the new position by hydrogen bonds to the side-chain O^γ^ and backbone NH of Ser139. Markedly, the oxyanion hole Gly143 NH, the correct positioning of which is essential for the stabilization of the tetrahedral oxyanion intermediate during catalysis, is moved 8.8 Å away.

**Figure 5:**
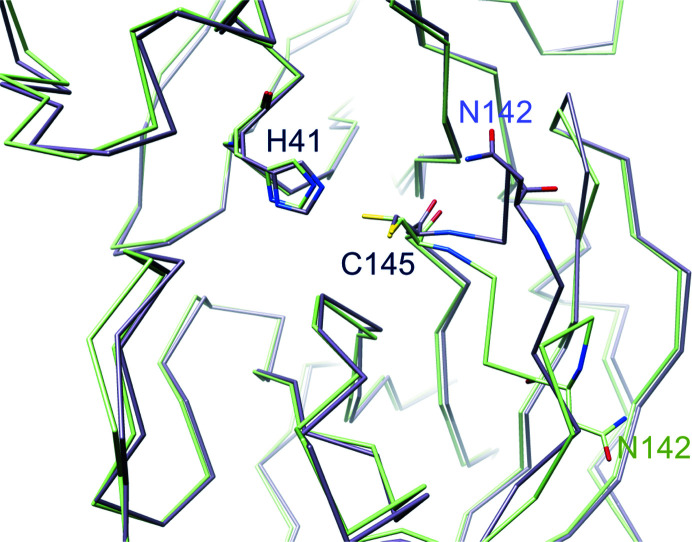
Catalytic dyad. In new-inactive M^pro^ (green) the position of the catalytic dyad His41 and Cys145 is similar to that in the active enzyme (PDB entry 6y2e, light magenta) despite the large shift of residues 138–144. In new-inactive M^pro^ Cys145 adopts a double conformation.

As a consequence, many interactions that are recognized to be important for stabilization of the active conformation are lost, namely hydrogen bonds between Glu166 and His172 and between Tyr161 and His163, as well as the aromatic stacking between His163 and Phe140 (Verma *et al.*, 2020 ▸). The rotation of the side chain of His163 (located at the very bottom of the S1 subsite), the hydrogen-bond properties of which seem to be very important in determining both substrate specificity and proper inhibitor binding (Deshmukh *et al.*, 2021 ▸), is a noteworthy characteristic of this new conformation of M^pro^. His163 is no longer available for substrate binding as it rotates away to avoid steric clashes with Gly143 CO (Fig. 4 ▸). Its position is now ‘functionally’ occupied by His172, which moves towards the S1 subsite (Fig. 4 ▸). The other three important residues, Tyr161, Met165 and Glu166, essentially maintain the same position as adopted in active M^pro^. Despite the large displacement of the oxyanion loop, the position of the catalytic dyad His41 and Cys145 is not significantly altered, especially in the backbone, even though the Cys145 side chain now shows a double conformation (Fig. 5 ▸). The conserved water molecule near His41 is still present in the same position, making hydrogen bonds to the side chains of His41, His164 and Asp187 as in active SARS-CoV-2 M^pro^.

### 3.4. The N-finger, the C-terminal tail and the dimeric interface are perturbed in new-inactive M^pro^

In new-inactive M^pro^, the dimeric interface is altered compared with that of the active conformation. *PISA* analysis of the interface shows that in new-inactive M^pro^ the interface area is reduced (from 1661 to 1273 Å^2^), as are the number of hydrogen bonds (from 33 to six) and the number of salt bridges (from 12 to six). However, structural features that are important for stabilization of the dimeric form are essentially conserved, namely (i) the salt bridge between Glu290 of one protomer and Arg4′ of the other (Anand *et al.*, 2002 ▸), (ii) the hydrophobic aromatic interaction between Tyr126 and Met6′ (Wei *et al.*, 2006 ▸) and (iii) the interaction of Arg298 with the N-finger and the C-terminus (Shi *et al.*, 2008 ▸). This suggests that although new-inactive M^pro^ is still able to form dimers, the dimeric state is less stable compared with that of active M^pro^.

**Figure 6:**
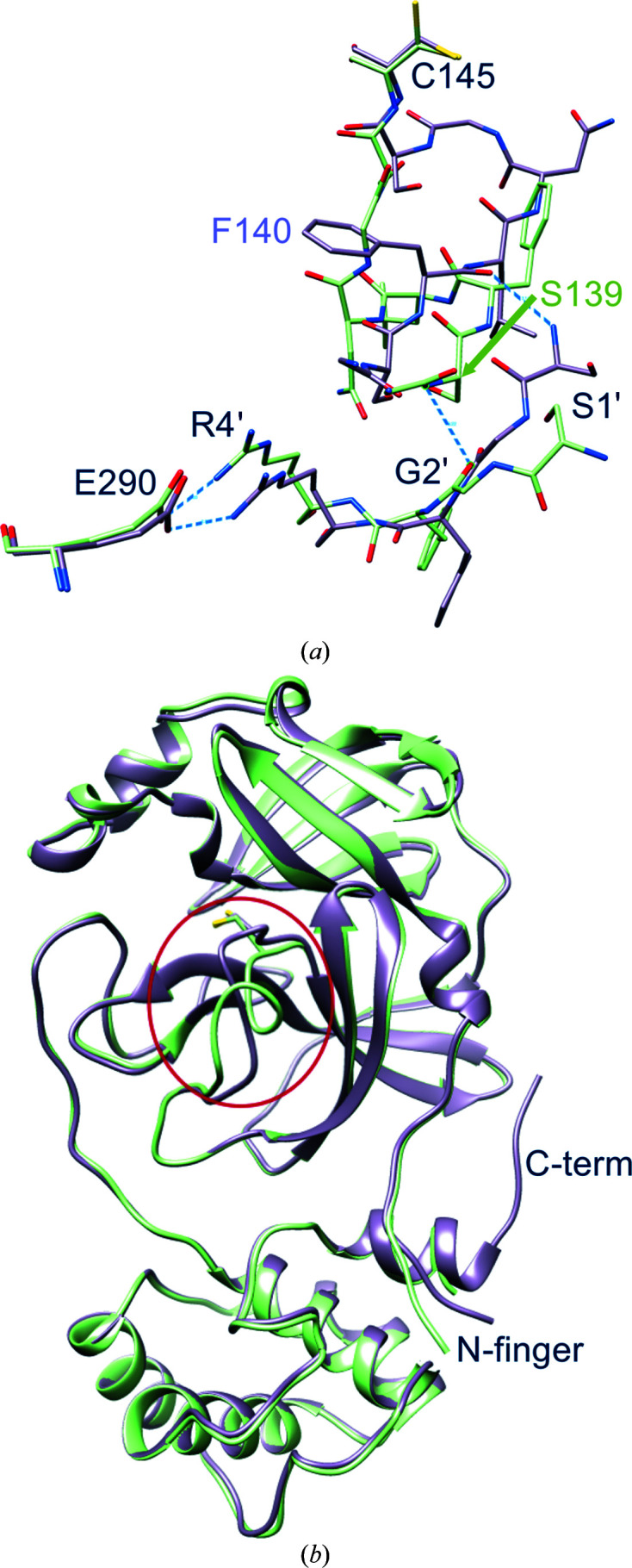
Displacements at the intra-protomer interface. New-inactive M^pro^ is in green and active M^pro^ is in magenta. (*a*) The new oxyanion loop of one protomer pushes away residues 1′–3′ of the other protomer; however, the key salt bridge between Arg4′ and Glu290, which is important for dimer stabilization, is conserved. (*b*) Overall superposition of active and new-inactive M^pro^ shows that besides those in the oxyanion loop (red ellipsoid), major differences are located in the N-finger and in the C-­terminal tail, which is not visible in new-inactive M^pro^.

**Figure 7:**
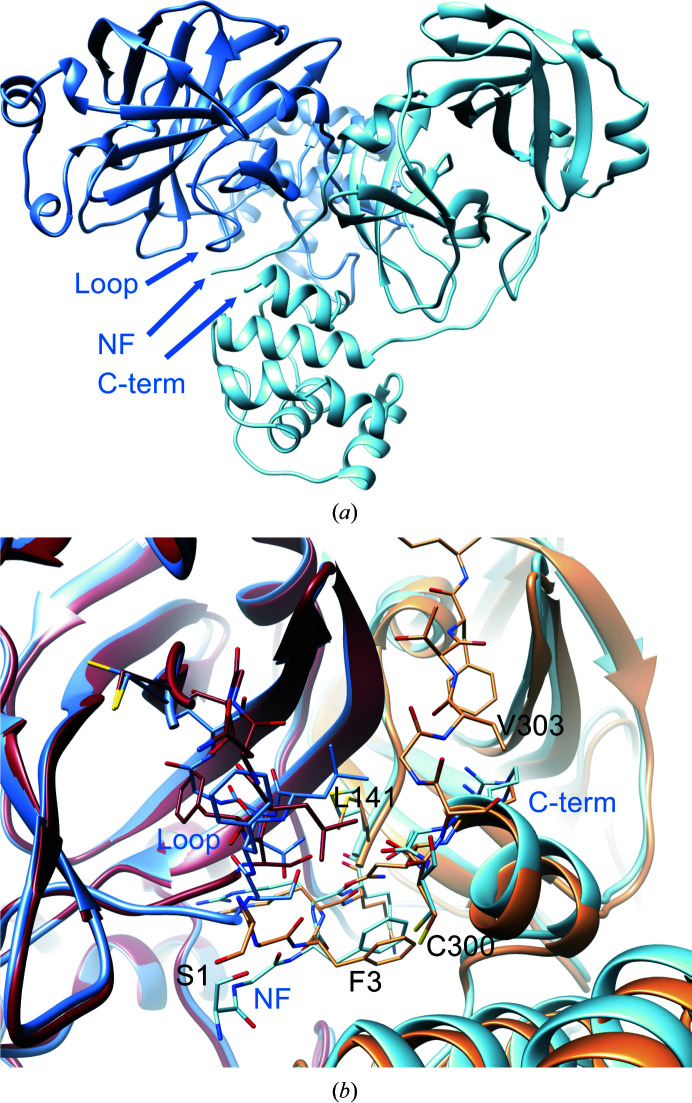
Dimeric architecture of new-inactive M^pro^. (*a*) The new conformation of the oxyanion loop (labeled ‘loop’) causes changes in the interface between protomer *A* (blue) and protomer *B* (light blue) at the level of the N-­finger (labelled ‘NF’) and the C-terminal tail (labeled ‘C-term’). (*b*) Local differences between the new structure [blue-based colors as in (*a*)] and the canonical structure (PDB entry 6y2e; brown-based colors, with intact C-terminus): the shift of the Leu141 side chain seems to have major effects in destabilizing the C-terminal tail of the new structure.

At the dimeric interface, relevant changes in both the N- and C-termini are present. In active M^pro^, the N-finger of one protomer interacts and stabilizes the S1 subsite of the other protomer (Verschueren *et al.*, 2008 ▸). For instance, in active SARS-CoV-2 M^pro^ (PSB entry 6y2e) Ser1 of one protomer is hydrogen-bonded both to the carboxylate group of Glu166 and to the main chain of Phe140 of the other protomer. In the new-inactive structure, these interactions are lost as a consequence of the different oxyanion conformation of one protomer that ‘pushes away’ residues 1–3 of the N-finger of the other protomer (Fig. 6 ▸), with Gly2′ CO now at 3.2 Å from Ser139 NH. The rearrangement of the oxyanion loop of one protomer also influences the C-terminal tail of the other protomer, the electron density of which is no longer visible from residue 301 onwards, indicating high flexibility (Figs. 6 ▸ *b* and 7 ▸). Among the residues of the oxyanion loop, Leu141 shows major changes at the level of the dimeric interface (Fig. 7 ▸ *b*), also causing rotation of the side chain of Tyr118 to avoid steric clashes, further supporting its possible central role in switching between the new-inactive and active conformations.

### 3.5. New-inactive M^pro^ can still bind substrates

**Figure 8:**
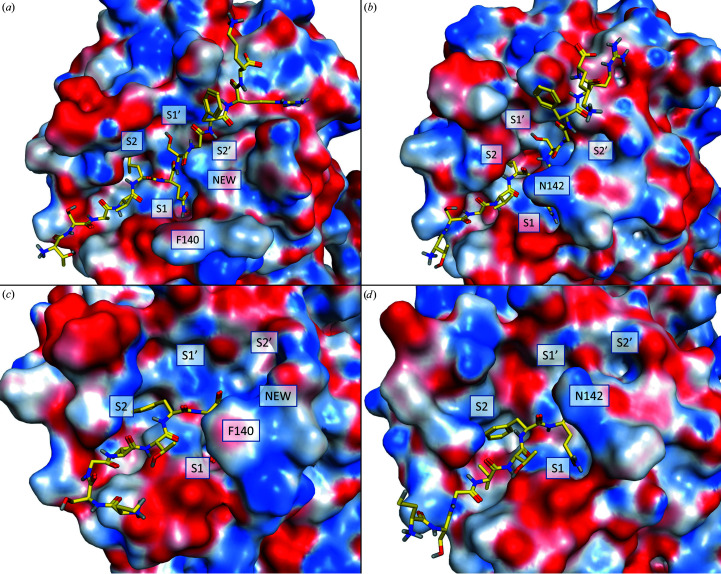
Reshaping of the S1 and S2′ subsites. Molecular-dynamics modeling of the hypothetical interaction of new-inactive M^pro^ with substrates is shown. Top, putative interaction with the 11-mer pseudo-substrate peptide from PDB entry 2q6g: (*a*) new-inactive M^pro^, (*b*) SARS-CoV M^pro^ from PDB entry 2q6g. Bottom, putative interaction with the acyl-intermediate of the M^pro^ C-terminal autoprocessing site: (*c*) new-inactive M^pro^, (*d*) M^pro^ in PDB entry 7khp. As a result of the rearrangement of the oxyanion loop, a new cavity near the S2′ site, labeled ‘NEW’, is formed.

**Figure 9:**
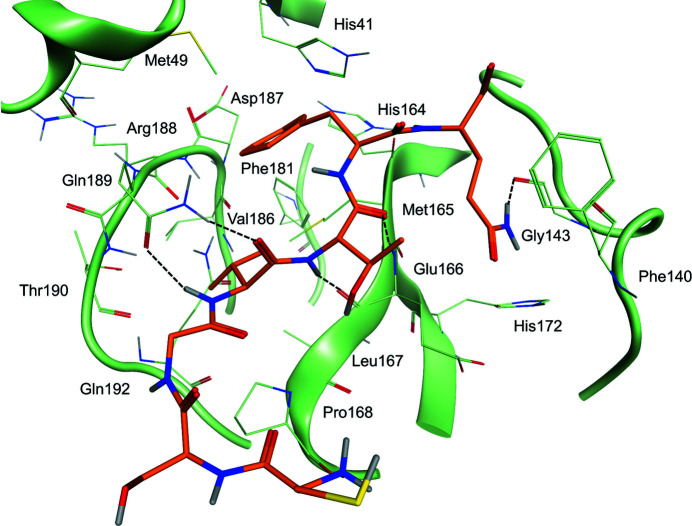
Details of the putative interaction between new-inactive M^pro^ (green) and the C-terminal acyl-intermediate peptide substrate from PDB entry 7khp (orange). Hydrogen bonds between the substrate and the binding site are depicted as dashed black lines. Aside from the P1 glutamine and its interactions with the P1 pocket, other common interaction features such as hydrogen bonds to Glu166 and Gln189 and hydrophobic interactions of the P2 phenylalanine side chain within the S2 subpocket are retained.

Having established that the new structure is catalytically incompetent, we tried to understand whether it is still able to bind natural substrates. Superposition of the new-inactive conformation with either the active conformation in complex with the C-terminal acyl-intermediate (PDB entry 7khp; Lee *et al.*, 2020 ▸) or the SARS-CoV M^pro^ active conformation in complex with its 11-mer substrate complex (PDB entry 2q6g; Xue *et al.*, 2008 ▸) does not show evident steric clashes for the substrate. This is also valid for superposition of the new-inactive conformation with two recent complexes between SARS-CoV-2 M^pro^ and two peptide substrates corresponding to the nsp4/5 (Kneller *et al.*, 2021 ▸) and nsp8/9 (MacDonald *et al.*, 2021 ▸) cleavage sites. Additionally, a short molecular-dynamics refinement of the complexes of the new-inactive conformation of SARS-CoV-2 M^pro^ with either the C-terminal acyl-intermediate or the 11-mer peptide substrate reveal compatible binding modes, with only minor side-chain re­arrangements (Fig. 8 ▸). The reshaped S1 site of the new-inactive M^pro^ could still host a P1 glutamine, although the rearrangement causes the loss of its interactions with Glu166 O^ɛ^ and Phe140 CO in favor of a single hydrogen bond to Gly143 CO (Fig. 9 ▸). Aside from the alterations of the S1 subsite, which alter the recognition profile of the P1 glutamine, the other interaction features are retained, namely the hydrogen bonds to Glu166 and Gln189 and the hydrophobic interactions of the P2 phenylalanine within the S2 subpocket. This is a quite remarkable observation because it suggests that the new conformation could be inactive not necessarily because it is incapable of recognizing the substrate, but because the catalytic machinery is not properly organized for an efficient catalytic event, particularly in the oxyanion-hole region, and is unable to stabilize the tetrahedral acyl intermediate. The new conformation of the oxyanion loop generates a new cavity near position S2′, as evident from comparison of the new structure with the SARS-CoV-2 acyl-enzyme (PDB entry 7khp; Lee *et al.*, 2020 ▸) and the SARS-CoV 11-mer substrate complex (PDB entry 2q6g; Xue *et al.*, 2008 ▸) (Fig. 8 ▸).

### 3.6. The new-inactive conformation is stable and is in equilibrium with the active conformation in solution

For SARS-CoV M^pro^, it has been shown that the active-site loops are very dynamic and sensitive to variations in the environmental conditions (Lee *et al.*, 2005 ▸; Tan *et al.*, 2005 ▸; Xue *et al.*, 2007 ▸, 2008 ▸; Yang *et al.*, 2003 ▸; Zheng *et al.*, 2007 ▸). Similarly, the oxyanion loop of SARS-CoV-2 M^pro^ showed conformational flexibility as deduced from room-temperature X-ray crystallography (Kneller, Phillips, Weiss *et al.*, 2020 ▸; Kneller, Phillips, O’Neill *et al.*, 2020 ▸). To test the stability and to model the dynamics of new-inactive M^pro^, specifically of the oxyanion loop and regions involved in substrate binding, we performed crystallographic ensemble refinement (Burnley *et al.*, 2012 ▸) and MD simulations.

**Figure 10:**
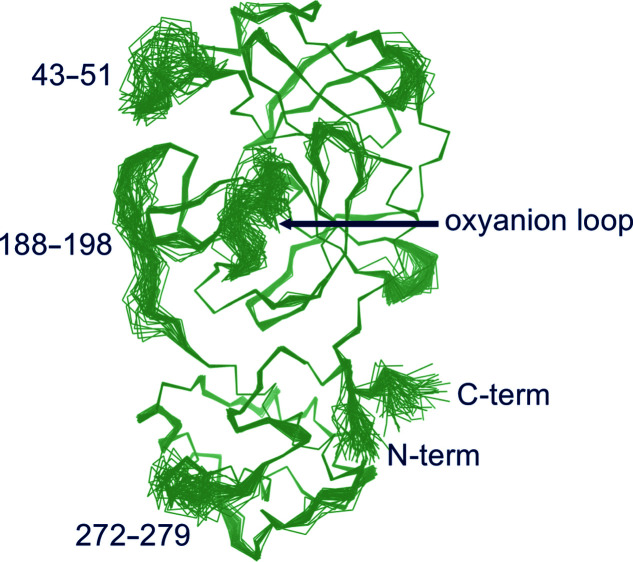
Ensemble refinement. The 60 structures generated by ensemble refinement highlight the mobile regions of new-inactive M^pro^. The oxyanion loop, which is confirmed in the new conformation, has a flexibility similar to those of residues 43–51 and 188–198 involved in substrate recognition as the S3 and S4 sites.

The 60 structures generated by ensemble refinement of new-inactive M^pro^ compatible with the crystallographic restraints confirm the new conformation of the oxyanion loop and reveal that its flexibility is comparable to that of other portions of the substrate-binding region (residues 43–51 in domain I and residues 188–198 in the flexible linker connecting domains II and III; Fig. 10 ▸), as also found in the literature. In four out of 60 structures the oxyanion-loop conformation is similar to that in the active form, which is in line with the experimental observation of a residual electron density compatible with the presence of a small fraction of the oxyanion loop and of the side chain of His163 in the active conformation in the crystal state. In this respect, all structures determined here, including new-inactive M^pro^, were obtained from batches of correctly autoprocessed protein (*i.e.* catalytically active towards itself at the N-terminus) which displayed normal catalytic activity in solution towards substrate peptides.

This strongly suggests the presence of a dynamic equilibrium in solution with the coexistence of different conformations, including inactive conformations. In other words, exhibition of the correct catalytic activity on the macroscopic level (with the full ensemble of conformational states available in solution for M^pro^) does not contrast with the possibility of selection by the crystallization process (in this case probably favored by the presence of certain small molecules) of a subpopulation of a catalytically incompetent form of the enzyme as shown here and for the previous structure with PDB code 1uj1. The conclusion that the dynamic equilibrium in solution includes both the active and the new-inactive conformation is supported by comparing the results of ensemble refinement of the structure in the free state with very poor electron density for the oxyanion loop (Supplementary Fig. S2*b* ). The refined ensemble conformations show a highly dynamic oxyanion loop, with 20% of conformations similar to the active conformation, 23% of conformations similar to the inactive conformation and 57% of conformations in intermediate states.

**Figure 11:**
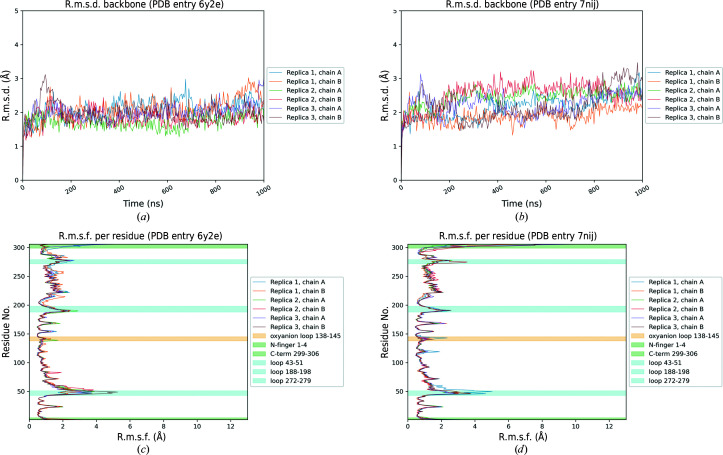
Results of MD simulations. Summary of the key geometric analysis performed along the MD trajectories for both the active (PDB entry 6y2e) and new-inactive (PDB entry 7nij) conformations of SARS-CoV-2 M^pro^. (*a*) and (*b*) highlight the time-dependent variation of the protein root-mean-square deviation (r.m.s.d.) of C^α^ atomic positions for PDB entries 6y2e and 7nij, respectively. (*c*) and (*d*) summarize the per-residue mean root-mean-square fluctuation (r.m.s.f.) of atomic positions of protein C^α^ atoms for PDB entries 6y2e and 7nij, respectively. The most relevant regions of the protein are highlighted in the plot for visualization clarity as described in the legend. For both r.m.s.d. and r.m.s.f. analyses, each chain composing the crystallographic dimer is considered separately.

To assess the structural stability of the new-inactive conformation of SARS-CoV-2 M^pro^ and to compare it with the active conformation, three independent 1 µs classical molecular-dynamics simulations were performed for both conformations. For the active state, PDB entry 6y2e was taken as a reference. As depicted in Fig. 11 ▸, which summarizes the principal geometric analysis performed along the MD trajectories, the two structures show a similar degree of stability. The backbone r.m.s.d. profile for PDB entry 7nij (Fig. 11 ▸ *b*), representing the new-inactive conformation of M^pro^, displays moderately higher fluctuations with respect to the active state (Fig. 11 ▸ *a*). As can be seen in the per-residue r.m.s.f. plots (Figs. 11 ▸ *c* and 11 ▸ *d*), this difference can mainly be attributed to major structural fluctuations in the same regions that were marked as flexible by the crystallographic data, namely the three flexible loops 43–51, 188–198 and 272–279 and the C-terminus (299–306), while the rest of the structure is quite stiff, as in the active state. Specifically, the C-terminus in the new-inactive conformation of M^pro^ shows the highest amplitude of movement, as denoted by the high r.m.s.f. values associated with these residues. This result agrees with the absence of electron density for residues 301–306, which indicates high flexibility of this region. Instead, the N-terminus (residues 1–4) shows more limited fluctuations for both M^pro^ conformations, which is in agreement with the presence of well defined electron density in both structures. The overall structural stability of the new-inactive conformation of M^pro^ is also confirmed by the time-dependent evolution of both secondary-structure elements and the protein radius of gyration (*R*_g_), with only minor oscillations, similar to those seen in the active conformation (Supplementary Figs. S4, S5 and S6). Despite the slightly higher fluctuations observed in the inactive conformation, no sufficient motions were observed to shed light on a possible transition mechanism between the two conformations. It is not surprising that such rearrangement was not sampled even on a 1 µs scale, since such collective motions in proteins usually involve longer timescales (*i.e.* millisecond to microsecond; Orellana, 2019 ▸).

## 4. Discussion

We had the opportunity to capture a new and stable (as seen in MD simulations) inactive state of M^pro^, called new-inactive, expanding the knowledge of the conformational space accessible to the enzyme. Altogether, the movements in the substrate-binding region and near the catalytic site result in a significant reshaping of the reaction center (Figs. 3 ▸, 4 ▸ and 8 ▸) that has never previously been observed and is much more pronounced than in the previously described collapsed-inactive M^pro^ conformation. The conformation adopted by residues 139–144 of the oxyanion loop is potentially catalytically incompetent. The backbones of key residues in the oxyanion hole are 8–10 Å away from the catalytically competent position. Fundamental interactions for the proper function of the enzyme are broken or absent, as illustrated in the previous section. Among the residues of the oxyanion loop, Phe140, Leu141 and Asn142 play a major role in the shift between the new-inactive and active conformations. The new state of the oxyanion loop of one protomer pushes the N-finger of the second protomer away from the position adopted in the active enzyme. The last six residues of the C-terminal tail are not visible in the electron-density map and were confirmed to be fully flexible by MD simulations. The novel conformations of the oxyanion loop and of the N- and C-termini result in a weakening of the dimeric architecture, as shown by decreases in the interaction surface area and in the number of inter-protomer interactions. Major variations in the dimeric interface are connected to Leu141 of the oxyanion loop.

This new structure is relevant for the analysis of the M^pro^ catalytic cycle, which was recently investigated using biodynamics theory under non-equilibrium conditions (Selvaggio & Pearlstein, 2018 ▸), using the available crystal structures, which show M^pro^ in different conformational states (Wan *et al.*, 2020 ▸). This novel approach tries to mimic *in vivo* conditions, which depend on non-equilibrium structure–kinetics relationships. From this analysis a substrate-induced M^pro^ activation mechanism was developed, suggesting the existence of a complex substrate-binding activation mechanism in both SARS-CoV and SARS-CoV-2. The proposed catalytic cycle involves transition from the collapsed-inactive conformation of the oxyanion loop, represented by the free form of monomeric M^pro^ (PDB entry 2qcy), to the putative substrate-bound form of monomeric M^pro^, represented by one monomer of PDB entry 2q6g (with an active oxyanion loop), and finally to the dimeric fully active state, represented by dimeric M^pro^ (PDB entry 6m03; very similar to PDB entry 6y2e). The new-inactive structure presented here shows a new conformational state with an accessible oxyanion loop, adding novel important pieces of information to the structural dynamics of the substrate-induced activation of M^pro^ in the context of its catalytic cycle. In the non-equilibrium model, it was hypothesized that transition of the oxyanion loop from the inactive to the active conformation is triggered mainly by solvation/desolvation effects. This also applies to transitions involving our new-inactive structure, where, for activation, Phe140 moves from an exposed position (with no minimally frustrated interactions) to a buried position (with eight minimally frustrated interactions), while Asn142 moves from a buried position to an exposed position. In the context of the conformational dynamics of M^pro^, the intriguing possibility esists that the remodeling of the S2′ subsite can be correlated with the large amino-acid variation in position P2′ of SARS coronaviral nonstructural protein (nsp) cleavage sites, M^pro^ autoprocessing included. Despite being catalytically incompetent, this new state (with a novel cavity in position S2′) seems to be able to bind natural substrates of M^pro^ (see Figs. 8 ▸ and 9 ▸). Among the 11 substrates of SARS-CoV-2 M^pro^, position P2′ is highly variable, hosting nine different amino acids with very different chemical and structural properties: small, such as Gly and Ala, bulky hydrophobic, such as Ile, Val and Leu, positively charged, such as Lys, negatively charged, such as Glu, and polar and hydrogen-bond donor/acceptor, such as Ser and Asn. It is conceivable that the flexibility of the oxyanion-loop conformation is correlated to this variability of the substrates, specifically in position P2′, and to the necessity to accommodate the different substrates during the maturation process of the pp1a and pp1ab polypeptides, in the correct succession of proteolytic events. We suggest that this new conformational state is that preferred by the enzyme to efficiently host substrates with bulky hydrophobic residues in position P2′, for instance for the processing of nsp7/8 (Ile), nsp12/13 (Val) and nsp14/15 (Leu) cleavage sites. According to the M^pro^ reaction scheme proposed by Wan *et al.* (2020 ▸), the substrate-binding event triggers the conformational switch of the oxyanion loop, which adopts the necessary conformation for a productive catalytic event. Overall, the following scheme can be proposed: (i) for the initial binding, specific substrates (with bulky residues in position P2′) select the new-inactive conformation among a complex ensemble of different conformations of M^pro^ in mutual equilibrium, (ii) the binding event causes conformational changes of the oxyanion loop and, mainly, of the side chains of Glu166, His172 and His163, (iii) the dimeric architecture is stabilized because of rearrangements of the N-finger and the C-terminus and (iv) the resulting activated enzyme is ready to properly hydrolyze the substrate.

The new-inactive structure is also important for the structure-based drug-discovery process that is currently being applied to M^pro^ (Deshmukh *et al.*, 2021 ▸). The approach of ‘repurposing’ already known drugs via classical docking methodologies on the 3D structure of the protein target is interesting because, methodologically, it is potentially fast and the safety profiles of the tested compounds are already known. This justifies the large amount of research devoted to repurposing known antiviral drugs against M^pro^ (Cannalire *et al.*, 2016 ▸). Obviously, the success rate of these campaigns would greatly benefit from the possibility of targeting significantly different, stable, conformations. In this respect, the discovery of the new stable inactive conformation of M^pro^ presented here, with the remodeling of the S1 subsite and the formation of the nearby new cavity near subsite S2′ (poorly explored until now as known inhibitors usually span the enzyme S1–S4 subsites), offers solid attractive possibilities for the design of completely new classes of antiviral drugs targeting M^pro^. Indeed, a putative binder of the new-inactive form could reduce the population of the active conformation by stabilizing the inactive conformation. Also, a ligand able to bind the novel, readapted site around the catalytic cysteine could sterically hamper the recognition of the substrate. In addition, the possibility of targeting a novel subpocket could increase the affinity by establishing novel contacts and interactions. Most of the more promising M^pro^ inhibitors were developed by optimizing starting hits that were further decorated to explore the sub­pockets located around the catalytic center, following the classic route of fragment maturation in fragment-based lead discovery (Yang & Yang, 2021 ▸). One notable example is represented by the optimization of portions of parampanel on S1 and S1′ and its engagement of S3–S4, which lead to a fourfold boost in IC_50_ activity (Zhang *et al.*, 2021 ▸).

In conclusion, the new-inactive structure of M^pro^ is relevant for better understanding of the function and mechanism of action of this fundamental enzyme for SARS-CoV-2 repli­cation in the cell, with a particular accent on the dynamics within the catalytic cycle of the enzyme, which explores different conformational states including that presented here for the first time. Further, the discovery of this unprecedented inactive conformation of M^pro^ provides a unique opportunity for the more successful design of antiviral drugs with improved pharmacological properties using both classical docking-based and innovative non-equilibrium-based approaches.

## Supplementary Material

PDB reference: SARS-CoV-2 main protease in a novel conformational state, 7nij

Supplementary Figures and Tables. DOI: 10.1107/S2059798322000948/ni5019sup1.pdf

## Figures and Tables

**Table 1:** X-ray diffraction data-processing and model-refinement statistics Values in parentheses are for the highest resolution shell.

Data collection	
X-ray source	ID23-2, ESRF
Wavelength (Å)	0.873130
Space group	*C*2
*a*, *b*, *c* (Å)	113.07, 54.71, 44.84
α, β, γ (°)	90.00, 101.30, 90.00
Resolution range (Å)	55.44–1.58 (1.61–1.58)
*R*_merge_	0.070 (1.305)
*R*_meas_	0.081 (1.505)
*R*_p.i.m._	0.040 (0.739)
Total No. of observations	145297 (7276)
No. of unique observations	36653 (1847)
Mean *I*/σ(*I*)	9.2 (1.0)
CC_1/2_ (%)	99.8 (35.7)
Completeness (%)	99.4 (99.5)
Multiplicity	4.0 (3.9)
Wilson *B* estimate (Å^2^)	23.7
Refinement	
Resolution range (Å)	55.44–1.58
*R*_work_/*R*_free_ (%)	17.71/20.31
No. of atoms	
Protein	2350
Water	218
*B* factors (Å^2^)	
Protein	32.6
Water	43.1
R.m.s.d.	
Bond lengths (Å)	0.008
Bond angles (°)	0.868
Coordinate error (maximum-likelihood-based by *Phenix*) (Å)	0.21
Ramachandran statistics	
Favored (%)	97.99
Allowed (%)	2.01
Outliers (%)	0.00
PDB code	7nij
Ensemble refinement	
No. of models	60
*R*_work_ */R*_free_ (%)	15.47/20.80
