# Supplementary material for: A new inactive conformation of SARS-CoV-2 main protease
Source: Acta Crystallogr D Struct Biol. 2022 Feb 21;78(Pt 3):363–78. doi: 10.1107/S2059798322000948 (PMC8900819; doi:10.1107/S2059798322000948)
Supplement: Supplementary file 1 [file d-78-00363-sup1.pdf]

STRUCTURAL
BIOLOGY

Volume 78 (2022)

Supporting information for article:

A new inactive conformation of SARS-CoV-2 main protease

Emanuele Fornasier, Maria Ludovica Macchia, Gabriele Giachin, Alice Sosic, Matteo Pavan, Mattia Sturlese, Cristiano Salata, Stefano Moro, Barbara Gatto, Massimo Bellanda and Roberto Battistutta

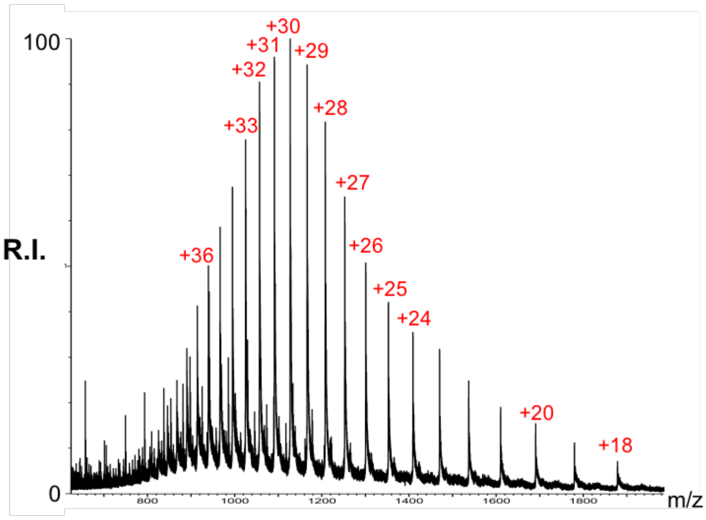

Figure S1

ESI-MS spectrum of full-length recombinant SARS-CoV-2 M^{pro} (residues 1-306). The sample containing 2 µM of the recombinant full-length SARS-CoV-2 M^{pro} was analyzed in 50% acetonitrile added of 0.1% formic acid in positive ion mode by direct infusion ESI-MS. For the sake of clarity, only a few charge states of the detected species are reported in the Figure. The species displayed a deconvoluted mass of 33796.64 Da vs a calculated one of 33796.81 Da.

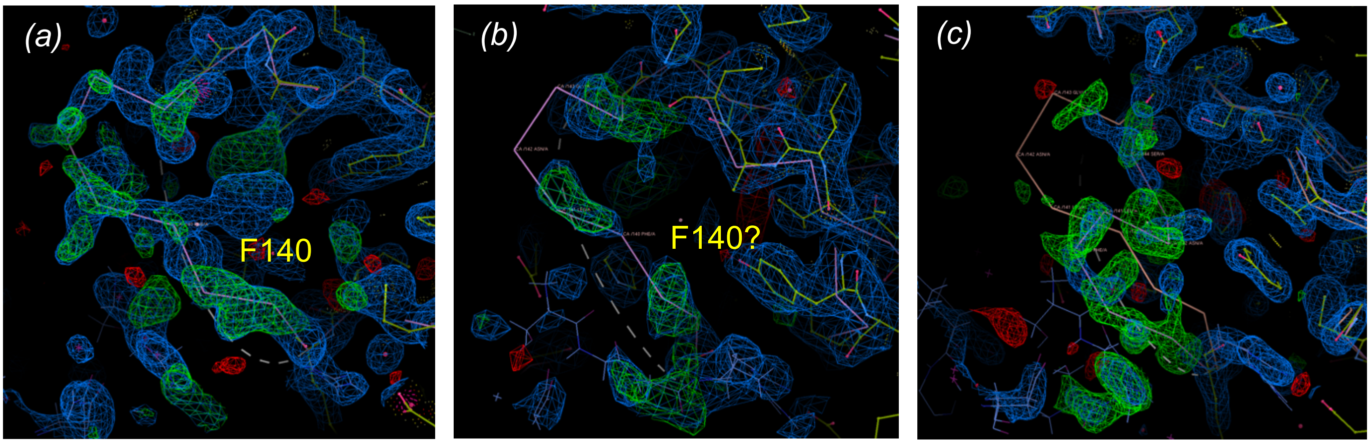

Figure S2

Initial electron densities. Representative situations after MR and a first step of refinement using a model lacking residues 139-144, 1-3 and with H163A substitution (see methods for details). Panel *(a)*, M^{pro} with good density of the oxyanion loop in the active conformation (Cα trace in magenta); side chains are visible, in particular for Phe140. Panel *(b)*, M^{pro} with very poor density for the oxyanion loop; no densities for side chains are visible. Panel *(c)*, M^{pro} electron densities clearly indicate a different conformation for the oxyanion loop (in orange the trace for the active conformation, in magenta the trace for the new conformation, new-inactive M^{pro}). 2Fo-Fc maps in blue (contour level 1.0 σ), Fo-Fc maps in green/red (contour level 3.0 σ). The white dashed line indicates the missing 139-144 residues.

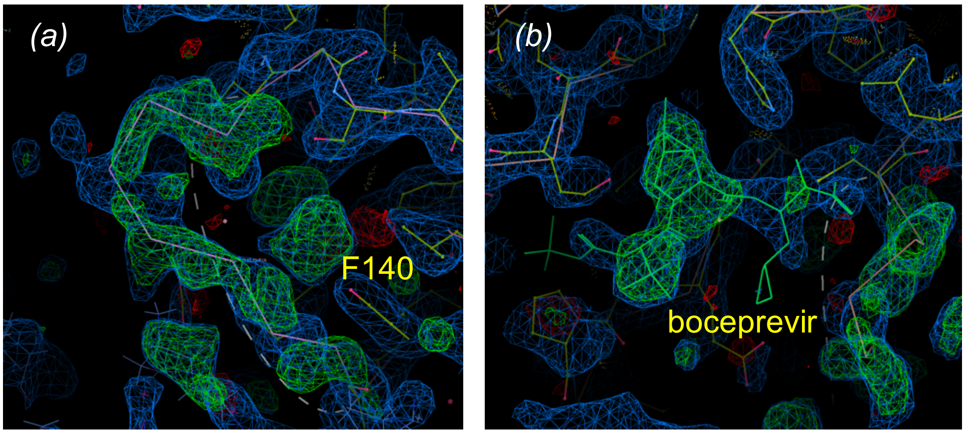

Figure S3

Initial electron densities for the M^{pro}/boceprevir complex. Representative situations after MR and a first step of refinement using a model lacking residues 139-144 and 1-3, with H163A substitution and without boceprevir (see methods for details). Panel *(a)*, M^{pro} with good density of the oxyanion loop in the active conformation (Cα trace in magenta); side chains are visible, in particular for Phe140. Panel *(b)*, electron density indicating the presence of the inhibitor (the boceprevir molecule, still not present at this refinement stage, is shown for reference). 2Fo-Fc maps in blue (contour level 1.0 σ), Fo-Fc maps in green/red (contour level 3.0 σ). The white dashed line indicates the missing 139-144 residues.

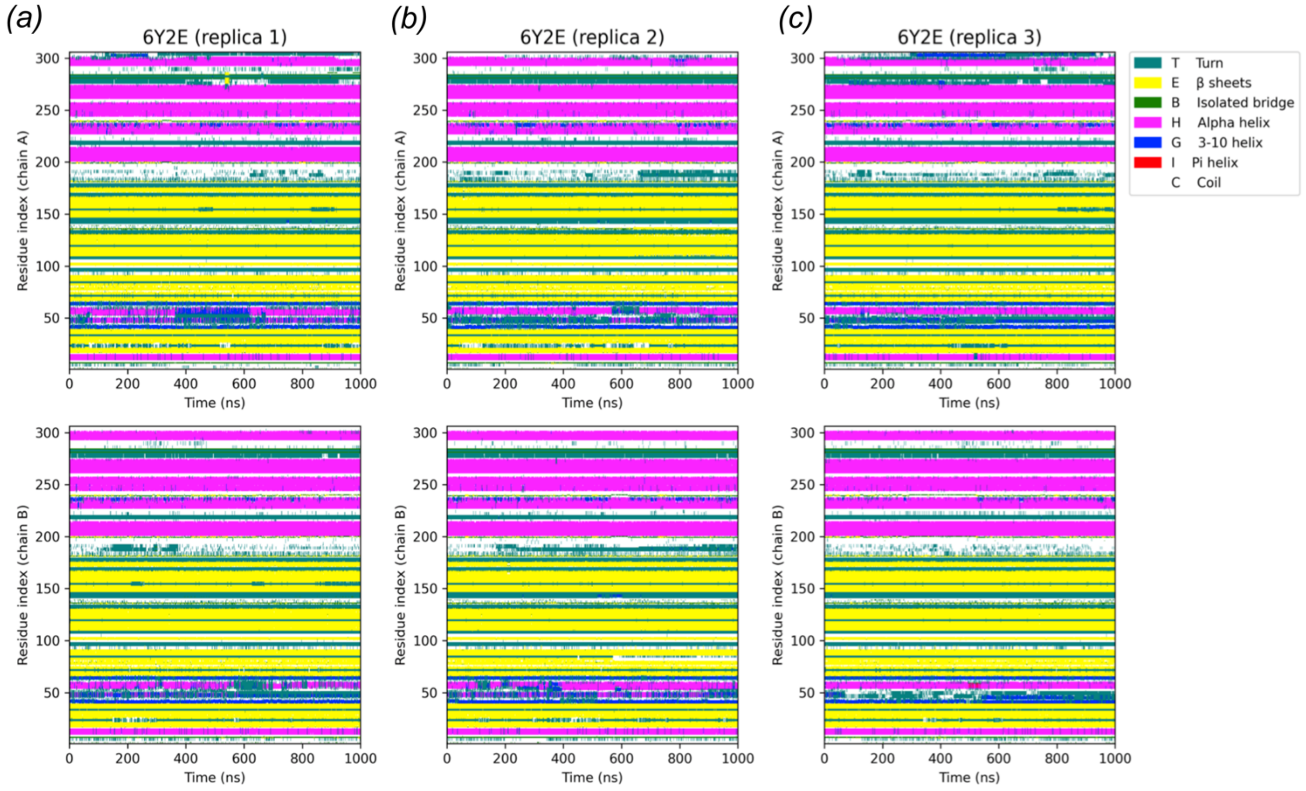

Figure S4

Time-dependent evolution of the secondary structure alongside the three MD simulations (*(a)*, *(b)* and *(c)*) for protein 6Y2E (active conformation). Colors are arranged according to Visual Molecular Dynamics (VMD) convention, as depicted in the legend. Within each subpanel, data referring to chain A of the dimer is reported in the upper part, while data concerning chain B is reported in the lower one.

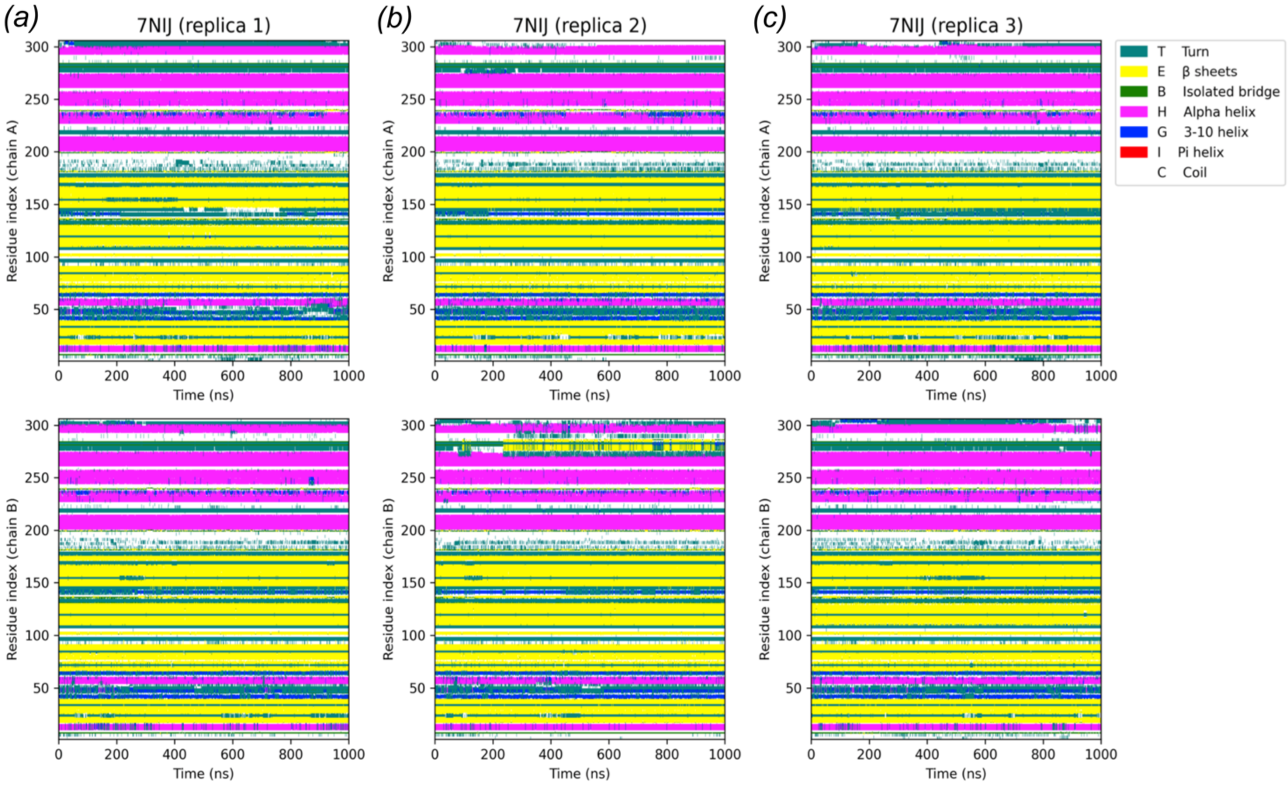

Figure S5
Time-dependent evolution of the secondary structure alongside the three MD simulations (*(a)*, *(b)* and *(c)*) for protein 7NIJ (new-inactive). Colors are arranged according to Visual Molecular Dynamics (VMD) convention, as depicted in the legend. Within each subpanel, data referring to chain A of the dimer is reported in the upper part, while data concerning chain B is reported in the lower one.

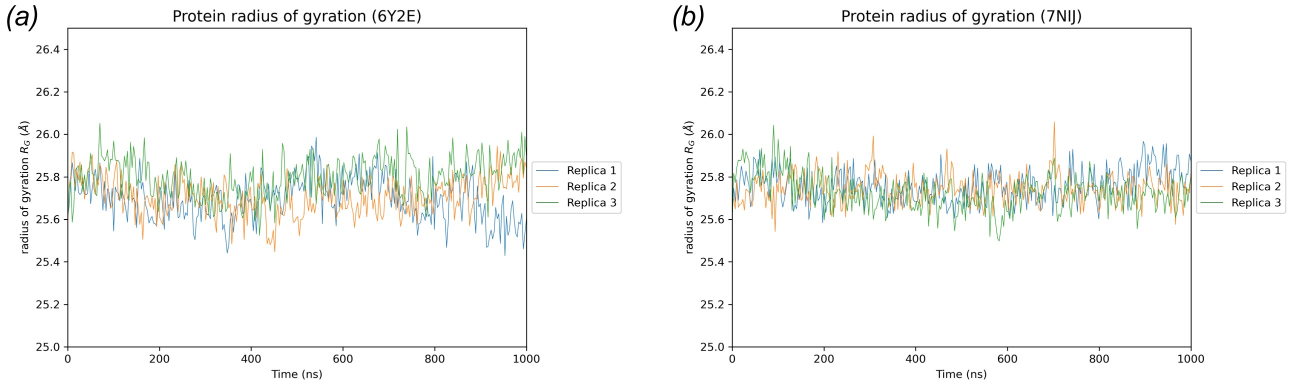

Figure S6
Time-dependent evolution of protein radius of gyration (R_g), for both the active (PDB ID: 6Y2E; panel *(a)*) and new-inactive (PDB ID: 7NIJ; panel *(b)*) conformations of SARS-CoV-2 M^{pro}.

Table S1
PDB codes and some significant pieces of information regarding the relevant structures of SARS-CoV-2 and SARS-CoV M^{pro} discussed in the paper. We used 6Y2E as reference structure for ligand-free, active SARS-CoV-2 M^{pro}. For recent comprehensive analyses of available SARS-CoV and SARS-CoV-2 M^{pro} crystal structures see Behnam, 2021; Brzezinski et al., 2021; Jaskolski et al., 2021; Wlodawer et al., 2020.
*Structure 7NIJ is presented in this paper.

PDB code	Coronavirus	Oxyanion loop conformation	Ligands	Mutations
7NIJ*	SARS-CoV-2	new-inactive	no	no
6Y2E	SARS-CoV-2	active	no	no
6M03	SARS-CoV-2	active	no	no
5REL	SARS-CoV-2	active	PCM-0102340	no
7K40	SARS-CoV-2	active	boceprevir	no
2BX4	SARS-CoV	active	no	no
1UJ1-B	SARS-CoV	collapsed-inactive	no	no
1UK2-B	SARS-CoV	collapsed-inactive	no	no
2QCY	SARS-CoV	collapsed-inactive	no	R298A
2Q6G	SARS-CoV	active	11_{mer} peptide substrate	no
7KHP	SARS-CoV	active	C-terminal acyl-intermediate	no

Table S2
Minimally frustrated energetic interactions involving selected amino acids of the active site in structure 6Y2E ("active") and structure presented in this paper ("new-inactive").

		6Y2E	new-inactive
**Cys145**	Tyr25	O	O
	Tyr26	O	O
	Asn28	O	-
	Gly29	O	O
	Cys38	O	O
	Val42	O	O
	Cys117	O	O
	Phe140	O	-
	Gly143	O	O
	Ser147	O	O
	Met162	O	O
	His163	O	O
	His164	O	O
	Met165	O	O
	Glu166	O	-
	Gly174	O	O
	**Total**	**16**	**13**
**Phe140**	Val114	O	-
	Ala116	O	-
	Ile138	O	-
	Cys145	O	-
	His163	O	-
	Met165	O	-
	Glu166	O	-
	His172	O	-
	**Total**	**8**	**0**
**Cys117**	Val13	O	-
	Glu14	-	O
	Met17	-	O
	Val18	O	O
	Gln19	O	O
	Leu27	-	O
	Gly29	-	O
	Val114	O	O
	Leu115	O	O
	Asn119	-	O
	Gly120	O	O
	Pro122	-	O
	Ser123	-	O
	Leu141	-	O
	Gly143	-	O
	Ser144	O	O
	Cys145	O	O
	Ser147	O	O
	Val148	O	O
	**Total**	**10**	**18**
**Leu141**	Ala116	-	O
	Cys117	-	O
**Gly143**	Cys117	-	O
	Tyr118	O	-
	Gly138	-	O
	Cys145	O	O
**Ser144**	Cys117	O	O
	**Total**	**3**	**6**
